# The complete chloroplast genome of *Paeonia lactiflora* Pall. (Paeoniaceae)

**DOI:** 10.1080/23802359.2019.1644548

**Published:** 2019-07-22

**Authors:** Minjee Lee, Jin Hee Park, Jinsu Gil, Jungho Lee, Yi Lee

**Affiliations:** a Green Plant Institute, Yongin, Korea;; b Nakdonggang National Institute of Biological Resources, Sangju, Korea;; c Department of Industrial Plant Science & Technology, Chungbuk National University, Cheongju, Korea

**Keywords:** Chloroplast, genome, medicinal plant, *Paeonia lactiflora*

## Abstract

The complete chloroplast genome sequence of *Paeonia lactiflora* was obtained by *de novo* assembly using next-generation sequencing. The circular molecule of the genome constituted of four parts, with 152,731 bp in the complete chloroplast genome, including a large single-copy (LSC) region of 84,402 bp, a small single-copy (SSC) region of 16,969 bp, and two inverted repeat (IRa and IRb) regions of 25,680 bp each. The genome annotation predicted a total of 111 genes, including 77 protein-coding genes, 30 tRNA genes, and 4 rRNA genes. Phylogenetic analysis showed the close taxonomical relationship to *P. veitchii*.

*Paeonia* (Peoniaceae) includes woody and herbaceous species. The germplasm of *Paeonia* species is important to the floral and medicinal industries, and *Paeonia lactiflora* has long been used in traditional medicinal in China, Korea, and Japan (Li et al. 2011; Zhou et al. 2019). The dried roots of *P. lactiflora* have been reported to be effective in anti-hyperglycemic, anti-inflammatory, and immunomodulatory applications (Hsu et al. 1997; Koo et al. 2010; He and Dai 2011). A genetic marker system for identifying *Paeonia* species has not been well established yet (Hosoki et al. 1997; Wang et al. 2009, 2014). We generated the complete chloroplast genome sequence of *P. lactiflora* to provide a genomic resource for basic genetic research and to help in the regulation of herbal medicinal plants through correct discrimination among the species in the genus *Paeonia*.

The leaves of *P. lactiflora* were collected from the Genetic Resource Research Greenhouse in Chungbuk National University (36°37′28.61″N, 127°27′18.36″E). The dried plant specimen (NIBRVP0000634118) was deposited in the Herbarium of the National Institute of Biological Resources (KB), Incheon, Korea. Total genomic DNA was extracted using a DNeasy Plant Mini kit (Qiagen, Valencia, CA, USA). The isolated genomic DNA was constructed to average a 600 bp paired-end (PE) library using the Illumina HiSeq platform. Contigs, assembled using a CLC Genomics Workbench (ver. 7.5, CLC Inc, Rarhus, Denmark), resulted in a total of 27.67 Gb high-quality PE reads collected in Illumina HiSeq NGS. The assembled structures and genes of the complete chloroplast genome were annotated using the DOGMA program (http://dogma.ccbb.utexas.edu/) (Wyman et al. 2004), and manually corrected based on a BLAST search. The *P. lactiflora* complete chloroplast genome sequence was deposited in the GenBank under accession no. MK860971.

The complete chloroplast genome of *P. lactiflora* was a circular molecule with a length of 152,731 bp, having 38.42% of GC content, which was composed of four distinct regions: a large single-copy (LSC) region of 84,402 bp, small single-copy (SSC) region of 16,969 bp, and a 25,680 bp in each of two inverted repeat (IRa and IRb) regions. We annotated 111 genes of the *P. lactiflora* chloroplast genome, including 77 protein-coding genes, 30 tRNA genes, and 4 rRNA genes.

In order to understand the phylogenetic relationship and genetic diversity between *P. lactiflora* and related species in *Paeoniaceae*, we performed phylogenetic analysis using the complete chloroplast genome sequence of *P. lactiflora* compared with those of 15 species, which included 12 ingroup species of the Saxifragales, to which *Paeonia* belongs, and three outgroup species of the Vitales (Figure 1). For the tree generation, we used the time-reversal model of the maximum likelihood algorithm with 1000 replications of bootstrap in CLC Genomics Workbench (ver. 11.0, CLC Qiagen). Six taxa of Paeoniaceae showed a monophyletic group and are separated into two clades containing three species each. *Paeonia lactiflora* belongs to the clade that includes *P. obovata* and *P. veitchii. Paeonia lactiflora* is close to *P. veitchii*.

**Figure 1.**
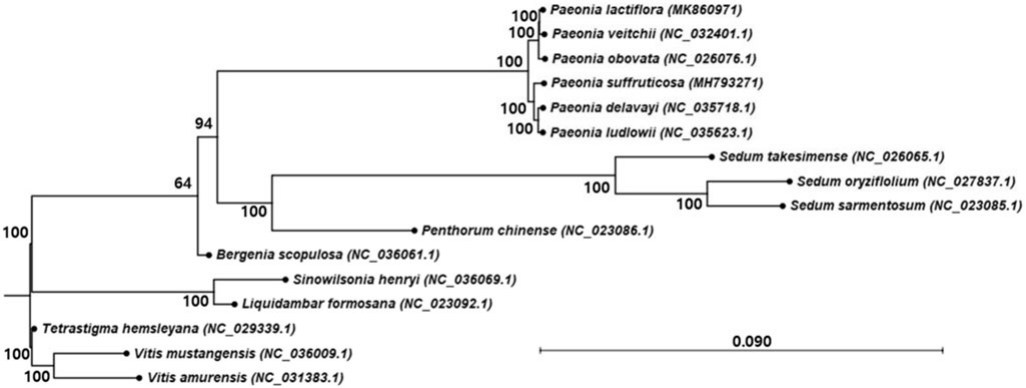
Maximum likelihood phylogenetic tree of *Paeonia lactiflora* with species in Saxifragales, to which the Paeoniaceae family belongs. The chloroplast sequences of the Vitaceae family were used as the outgroup. Numbers in the node are the bootstrap values from 1000 replicates.
